# Exploring Cognitive Processes of Knowledge Acquisition to Upgrade Academic Practices

**DOI:** 10.3389/fpsyg.2022.682628

**Published:** 2022-05-06

**Authors:** Deepa Cherukunnath, Anita Puri Singh

**Affiliations:** ^1^Indian Council of Social Science Research, New Delhi, India; ^2^Department of Psychology, Government M L B Girls PG College, Bhopal, Bhopal, India

**Keywords:** learning environment, cognitive skill enhancing activities, knowledge acquisition, curriculum development, cognitive skill development

## Abstract

The development of cognitive functions follows certain pathways through brain maturation. Concepts taught at school can be reinforced by understanding the related cognitive functions that enhance learning. The cultural and social diversities faced by the education system worldwide can be solved by understanding the unifying cognitive processes of learning. This knowledge can be effectively used to devise better curriculum and training for students. Cognition, conation, and emotional regulation are the main components that determine an individual’s efficiency to deal with various situations. How the brain receives input, perceives, and organizes these information lays the foundation for learning. The objectives of the study were (i) to explore age-group specific inputs for knowledge acquisition, (ii) to relate knowledge organization to the cognitive processes, and (iii) to identify factors that strengthen the knowledge ensemble through subject-domain allied training. The review focused on studies related to elementary school age (below 7 years), middle school age (7–12 years), and high school age (12 years and above). Published journal articles related to the objectives were randomly reviewed to establish a possible relationship. The findings of this review can help to advance student learning practices and instructional strategies. The findings are listed below. (i) Acquisition of knowledge during early childhood is based on sensory-motor integration on which attentional, perceptual, memory, language, and socialization systems develop. As brain development progresses toward adolescence, meta-awareness and social-emotional cognition influence the student learning process. (ii) Knowledge representations can be strengthened by domain-specific training inputs. (iii) Associational integration of the developmental, cognitive, and conative processes are indicators of curriculum strength. (iv) The strengthening of cognitive processes by rerouting through complementary neural circuitry, such as music, arts, real-life-based experiments, and physical exercises, is an effective way to improve child-friendly instructions.

## Section 1: The Background: Developmental Processes Related to Knowledge Organization

Our education system is based on the processes of learning that emphasize the reciprocal interaction between biological and environmental influences. The period of preschool to adolescence is marked by intensive psychological changes associated with central nervous system maturation. Learning concepts is one of the main developmental tasks that coincide with this period. It includes cognitive, behavioral, and conative factors. Cognitive functions are the framework on which a knowledge system develops, whereas conative factors like motivation and emotional state modulate the learning behavior. Understanding these functions can support the processes of learning, teaching, and making policy changes in education (Varma et al., 2008).

The learning process brings changes related to the ability, creativity, flexibility, and self-regulation of an individual. Schematic knowledge representations acquired by experiences make every human brain unique. Fischer and Bullock (1984) suggested that macro developmental changes, such as growth and maturation, are universal, while micro developmental changes, such as individual differences, are individualistic. Efforts made by Epstein (2001) helped to stratify the development of milestones based on vertical and horizontal decalages for formulating training strategies. Advances in imaging techniques have provided more insights about the development of brain circuits at the cellular, molecular, and structural levels. Normal development of brain circuits is influenced by genetic predisposition, environmental situations, and neuroplastic response to experiential demands (Tau and Peterson, 2010). Cognitive abilities and related behavioral regulations are developed within these circuits, so also are knowledge systems. This article reviews the developmental aspects of brain maturation that enable us to understand knowledge acquisition in school students. The age-wise cognitive requirements are described to frame the curriculum for providing effective training.

The objectives of the study were to explore knowledge organization by review of research related to influences of environment on the acquisition, mapping of knowledge organization, and methods to advance academic practices. Knowledge acquisition for the purpose of this study consists of experiences and the conceptual framework required for incorporating new information. School curriculum can be enhanced by making the knowledge acquisition process more favorable to students. To understand these processes, the information has been compiled under the following subheadings in this section: (a) Influences of environment on brain development, (b) knowledge organization, and (c) upgrading academic practices based on knowledge organization. Scientific articles published in peer-reviewed journals and textbooks were randomly referred for this purpose.

### a. Environment and Its Influence on Brain Development

The uniqueness of every individual depends on the approach of perceiving information present in the environment and its synchronized processing at the cognitive level. The environment and experience exert a major influence on the brain functions related to knowledge development. The cumulated impact of biological preparedness (experience-expectant plasticity) and conducive environment (experience-dependent), which includes biological maturation and educational experiences, respectively, affects learning (Goswami, 2008). Studies indicate that the development of neural circuits depends on environmental stimulation during sensitive periods (Tau and Peterson, 2010). The interaction of multiple brain systems related to concept learning mechanisms adapts category structures and motivational states that are modulated by experiences (Zeithmavo et al., 2019).

These sensitive periods are critical to prove the dependence of experience on plasticity. New synapses are formed based on varied experiences that strengthen the brain circuits. Cortical thinning, myelination, and experience-dependent remodeling of neural circuits are known factors that predict cognitive functions. Maturational trajectories for affective development and its regulation can be extended through enriching experiences, unlike the other sensory motor systems (Hartley and Lee, 2015). The human brain creates knowledge by integrating cues with social context (Bolisani and Bratianu, 2018). The physical, cognitive, psychosexual, and moral maturation determine a child’s future adaptability in the environment (Volkmar and Martin, 2009). Exposure to chronic stress accelerates cellular aging and faster maturation due to repeated use of stress detection and stress regulation circuitry (Tooley et al., 2021).

Psycho-social theories also support the role of the environment on cognitive development. Vygotsky emphasized on the “cognitive cultural heritage” that the child receives from the adults (Tomic and Kingma, 1996). Social cognitive theory (Bandura, 1970) explained how events are perceived, interpreted, and learned by an individual. Learners are hence predisposed to learn incidentally, explicitly, or implicitly through the influence of learning conditions (Robinson, 2011).

The role of environmental stimulation on the sensory integration process, language stimulation, behavior modeling, brain maturation, and above all, the cognitive cultural heritage is well established. The emergence of functional networks corresponding to environmental demands is an interesting idea that can be researched in the future.

### b. Knowledge Organization

Inputs related to knowledge organization require an understanding of the neural activation through multi-sensory experiences, development of motor and language, and social–emotional adaptability. The curriculum consists of concept knowledge and related tacit knowledge. Conceptual knowledge is the relational representations of core principles about general and abstract knowledge (Schneider et al., 2011), whereas tacit knowledge is implicit and includes attitudes, feelings, and experiences that define how one learns to behave in various situations. Object knowledge is stored as sensory-derived knowledge or language and cognition-derived knowledge as testified in congenitally blind individuals. Abstract concepts are stored in associated sensory, emotional, and complex event experiences (Wang et al., 2020). Most theories support the view that networks for space, time, and number are intertwined at birth. The relation between space and number that is observed in a new-born child is found to have a high correlation with motor behavior and visual system (McCrink and Opfer, 2014). The interaction of visual pathways and limbic system aids in visual recognition memory (Kotrla and Weinberger, 2000). Primary sensory inputs in the form of auditory and visual stimuli play an important role in the development of attentional and perceptual processes (Ackerman, 1993), which is the foundation for creating phonological awareness in children. Stimulation during critical periods of development influences cortical organization and plasticity. Synaptogenesis happens only later in the anterior regions of the brain, including the prefrontal cortex (Huttenlocher, 1984). Tau and Peterson (2010) functionally analyzed the development of neural circuits during late childhood and adolescence and reported its influence on self-regulation and cognitive control.

Every activity forms a corresponding network of patterns that creates sensory inputs, thoughts, emotions, and motor responses (Bouchard, 2005). Knowledge networks are thus mapped based on the representations formed during the early developmental phases. Neural circuits subserving primary sensory-motor areas mature earlier, followed by attentional processes, and socio-emotional functioning occur at a later stage.

Bottini and Doeller (2020) identified that conceptual knowledge is organized in hippocampus-based knowledge frames that provide inputs related to world-centered representations (cognitive maps). Self centered spatial schemas based in parietal cortex are body centered, sensory-motor grounded, and attention mediated high level cognition. These two representations play a crucial role in goal-oriented cognition, analogical thinking, generalization, and structuring of abstract conceptual domains. Time, number, valence, and socio-emotional ties are low-dimensional ego-centric schemas derived from sensory-motor experiences (Bottini and Doeller, 2020). Schemas are relational knowledge that connects the real-world higher cognition in a logical sequence by providing an integrative framework for inference, categorization, quantification, planning, language, working memory, and knowledge acquisition (Halford et al., 2010). Researches indicate the strength of functional connectivity of frontal, parietal, occipital, and limbic lobes to the integration of information (Semrud-Clikeman, 2010).

The knowledge schemas are representations stored in ways as perceived along the developmental course. Academic concept networks are formed on the relational association that includes biological predisposition, stimulation from the external world, and formation of concept-linking functions. Hence, structuring knowledge acquisition and organization through the curriculum will define its utilization.

### c. Upgrading Academic Practices Based on Knowledge Organization

Cognitive processes related to academic concept formation are acquisition, assimilation, generalization, and association of information. The role of sensory-motor integration, spatial encoding, and associating to existing networks are the main adaptive features related to academic learning. Thomas et al. (2019) summarized eight interacting learning systems in the brain, namely memorizing episodic and autobiographical memory, perceptuo-motor areas for spatial and temporal patterns, classical conditioning associations, integration of planning and emotions, the reward-based systems, procedural learning systems, learning by modeling, and language construction systems.

Enhancing aptitude in academics can be gained by nurturing specific cognitive skills in students. Curriculum that aids in sharing the core cognitive elements between tasks can facilitate the transfer of learning. Information related to training students, discussed below based on research conclusions, bring more insights. Activities linked to core identifying factors like gross and fine motor representations and music training, body kinesthesis, creative writing, and arts can improve concepts in math and science. Relating the concept of musical rhythm with spatial cues of time, prosody, aesthetics, symmetry, and emotions is found to improve numerical cognition in developmental dyslexia (Ribeiro and Santos, 2020). Children who have undergone early music training before 10 years of age are found to have a high degree of sensory-motor integration that can enhance plasticity during the sensitive period (Penhune, 2011). Involving students in these activities will improve their academic skills in math, science, spatial ability, problem-solving, and logical understanding. Exposure to sound rhyming patterns in early years improved phonological development and reading fluency during school years (Goswami, 2008). The role of experience in human development is to integrate the similarities in the streams of information through exposure, linguistic prompts, and motor plans. Focus on domain-general cognitive processes, which includes logical reasoning, heuristics, and problem-solving strategies, develops creative thinking in school children (Newcombe et al., 2009).

Math training based on conceptual understanding-based strategies enables more effective changes in students over fluent training of facts and procedures (Rosenberg-Lee, 2018). Despite the curriculum for teaching math and science skills in higher classes following the activity-based approach, the associative learning that occurs mostly gets hindered due to the process of habituation and sensitization caused by a reduction of the novelty factor (Çevik, 2014). Rote and repeated task practice resulted in accelerated maturation of specific brain circuits. Children exposed to variable experiences are found to benefit by the delayed brain maturation process (Tooley et al., 2021). The transfer of learning takes place only when the activities are designed according to the novelty that corresponds to the age and maturation level of the student. The phenomenon of blocking interferes when students are overexposed to conditioned activities that reduce the reinforcement value (Papini and Bitterman, 1990). Repetition of the same threshold evoking activities followed in curriculum causes reduction in stimulation of sensory neurons. Activities that include distal cues by relating to life situations and adaptive skills can increase curiosity. Also, stimulation that corresponds to the expansion of more synapses makes learning stronger. At the neuronal level, repeated coactivation of two neurons leads to long-lasting synapses, which provides the substrate for long-term memory (Owens and Tanner, 2017).

The main components that enhanced adaptability while training on executive functions were updated working memory representations, increased cognitive flexibility, and inhibiting competing responses (Diamond, 2013). Most recent research opinions on direct working memory training do not promise transfer effects to the academic domain during childhood and adolescence. Neuroimaging findings of children and adolescents suggest shared neural substrates between motor and working memory tasks and recommends the promotion of motor skills and functions as a pathway to improve working memory (Ludgyga et al., 2022). Executive functions are related to real-life goal-directed behaviors (Pluck et al., 2020). Curriculum-integrated training of related cognitive processing through activities indirectly strengthen the subject-domain network. Cognitive skills are strengthened by involving students in supplementary activities, such as music, arts, and sports. Hence, training plays a major role in providing varied experiences. The findings mentioned above provide insights on the benefits of understanding the acquisition and generalization of information.

## Section 2: Objectives of the Review

The objectives of the review are as follows: (i) to explore environmental inputs for knowledge acquisition, (ii) to relate knowledge organization to the cognitive processes, and (iii) to identify factors to upgrade academic practices.

The review focused on studies related to elementary school age (below 7 years), middle school age (7–12 years), and high school age (12 years and above). Scientific articles published in peer-reviewed journals and textbooks were randomly referred for this purpose.

## Section 3: Training Recommendations Based on Cognitive Development

Developmental neuroscience research generates valuable insight into specific age-related neural characteristics and processes. The development reflects the interaction of a child’s neurological system with the environment in a predictable sequence, and studies indicate that maturational changes due to development are independent of training (Klingberg, 2014). Thus, the survival circuits are formed initially, followed by knowledge acquisition, and then self-awareness and meta-cognition. This article suggests age-wise developmental stimulation from elementary age to adolescence.

### Elementary School Age (Below 7 Years)

Visual, auditory and somesthesis development occurs parallelly during this phase. Association of these sensory stimuli represent the first level mental constructs (Epstein, 2001). This period is critical for the socialization and its related skills, including the development of language (both lexical and syntax), face recognition, and motor development (Tierney and Nelson, 2009). The integration cues adopted by children to learn language during the first 4 years of life help to control and construct a representation of events (Ramscar and Gitcho, 2007).

The focus of strategies for training preschool children should be on the causal-logical structure of the content deciphered, by discussing the goals, consequences, and themes (Kendeou et al., 2007). Deliberate learning practices are to be adopted to foster attention skills. Attention skills during this phase are inhibitory, which allow the child to focus on targets, often resulting in difficulty to disengage the attention toward other activities (Ackerman, 1993). Inputs from sensory information systems, confidence in the use of motor movements, understanding body kinesthesis, perceptions, and newfound ability to relate thoughts are the functions that can be nurtured from early childhood. Involvement in fine and gross motor activities, observation, and enriching emotional experiences provide a better platform for development. Emotion is a primary function of the central nervous system to adapt behavior according to environmental conditions. Emotion heightens the salience of environmental features during sensitive periods to enhance the sensory experience, and that orients the organism towards the developmental challenges (Nelson et al., 2014). Li et al. (2020) recognized that various facets of learning, including attention, motivation, memory, and social interaction, are connected to emotions.

Reciprocal teaching helps students acquire specific knowledge and strategies for reading, writing, and arithmetic skills by explicating, elaborating, and monitoring for independent learning (Doolittle et al., 2006). Rivière (2014) conducted experiments in infants and toddlers to conclude that decision-making processes in tasks involving motor responses can be attributed to the embodied-cognition approach.

### Middle School Years (7–12 Years)

During the middle school years, the focus shifts to widening of academic concept development. Refining the learning techniques that focus on the self-regulatory capabilities of students of higher elementary classes are found beneficial over the content (Dunlosky et al., 2013). Frontal lobe connections get strengthened with more stimulation, especially in the prefrontal regions (Semrud-Clikeman, 2010). With the widening of association areas, academic skills become stronger through assimilation and adaptation of learned information. Connections of the frontal area to the emotional areas and related memory pathways strengthen the deeper understanding of concepts. Associational learning using logical reasoning to generalize past experiences is a prominent methodology adopted by this age group. van Kesteren and Meeter (2020) listed effective memory methods like elaboration, linking of existing schemas through associative iterations, and proving distinctiveness of stored information with episodic details to increase school performance. Innovative practices in subject combination, for example, linking numerical series with musical prosody and poetry syllable appreciation, help in knowledge-related aptitude formation. An integrated cognitive approach, as mentioned, can be effectively applied to generate novel opportunities to interconnect various cognitive tasks. Multi-sensory integration facilitates transient synchronization of brain operations as it combines various signals to form a new multimodal representational percept (Fingelkurts et al., 2005). Long-term music training enhanced number production, number comprehension, number line, and calculation in children with dyscalculia (Ribeiro and Santos, 2020). Executive function skills training provided during childhood indicated far transfer effect in maths and its related changes in cognitive functions (Zelazo and Carlson, 2020).

### High School Years (12 Years and Above)

Development of academic functions during adolescence becomes more analytical, organized, and relational. Increased autonomy to choose experiences differentiates this stage of development from the structured environmental demands of childhood. Self-determination theory (Ryan and Deci, 2000) highlights the inherent resources for behavioral self-regulation. Ryan and Deci identified three psychological needs – competence, autonomy, and relatedness that foster self-motivation and personality integration. Teachers and parents who support autonomy could catalyze the student’s intrinsic motivation. Cognitive functions, including metacognition, intelligence, working memory, problem-solving, and social cognition, are the major functional changes observed during adolescence (Fuhrmann et al., 2015). The method by which information is received and perceived determines how it will be processed. Li and Jeong (2020) reported that learning a second language in adults is effective when the focus of learning is shifted to the social interactive brain. The emerging methods of learning language consisted of social interaction with people and objects, whole-body embodiment in the learning context, self-exploratory learning, and sensory-motor integration. So, students with learning difficulties can utilize social and affective trajectories to enhance their learning experience.

Adolescents are more motivated by rewards than adults, less averse to risks, and easily influenced by peers (Steinberg, 2005; Tau and Peterson, 2010). Self-regulatory and reward systems participate together in the resolution of conflicts between the intrinsic hedonic value of incentives and societal consequences. Guyer et al. (2018) reported heightened brain activity elicited in adolescents explicitly related to reward cues, salient emotions, and peer influence as characteristic to this age group. The curriculum should emphasize social skills for inculcating interpersonal and intrapersonal relationships, along with contextual training, to achieve the selective goal.

Findings are conclusive that the focus of student cognitive skill training during early childhood should be on sensory-motor integration, socialization, and cognitive control that facilitates knowledge acquisition and assimilation of primary constructs. Middle school period latency promotes functional integration through generalization and association. High school years initiate impetus for autonomy by gaining insights on meta-cognitive and social-emotional control.

## Conclusion

Recently established research practices of utilizing technologically aided non-invasive strategies to understand brain maturation and related mapping of cognitive abilities can advance student learning practices and instructional strategies. Factor analytic identification of domain-specific cognitive processes helps to strengthen knowledge-related clusters that can determine student aptitude in various academic fields. For example, critical thinking and analytical approach in science can be enhanced through strengthening the associated cognitive processes by involving students in supplementary arts, music, and body kinesthesis, which creates the same synchronization at the neural level.

Academic skills require synchronized functioning of complex cognitive skills. Since school years coincide with the widening of neuronal connections to various areas, designing of curriculum and pedagogy needs to incorporate inputs from the fields of developmental cognitive psychology. Acquisition of knowledge during early childhood is based on sensory-motor integration on which attentional, perceptual, memory, language, and socialization systems develop. As the development progresses toward adolescence, the emergence of higher cognitive and social-emotional cognition influences the learning process. Academic skills are generalized associations of afferent and efferent systems that organize the schema representations.

Knowledge representations can be strengthened by providing domain-specific training. The phenomenon of transfer of learning relates domain-specific skills to make a positive or negative effect on various personal abilities (Ramscar and Gitcho, 2007). Factors that aid in the transfer of learning are context, problem representations, shared cognitive elements between the tasks, and metacognition (National Research Council, 2000). These are functions of the cortico-striatal loop that combines sensory-motor, associative, and limbic zones, which show improvement with cognitive training (Klingberg, 2014).

Research proves that classroom instructions that are not appropriate for a child’s level of maturation lead to behavior problems like avoidance, challenging authority, and aggression (Semrud-Clikeman, 2010). Strengthening compensatory or strategic processes by rerouting through complementary neural circuitry, such as music and arts, are effective instructional remedies for cognitive training. Music and physical exercises thus act as compensatory remedies. The students facing difficulty in math or language due to lack of environmental stimulation may benefit through these compensatory trainings. Relational integration of all these concepts is a strong indicator of school performance that gets integrated through fluid reasoning ability (Bryck and Fisher, 2012).

Improving equity in the educational environment can be achieved by upgrading interventions linked to domain specific skill training and by providing enriching environmental exposure during the sensitive periods (Sokolowski and Ansari, 2018). Further experiments in this research field can be designed to cluster-specific brain functions related to various academic domains, thus identifying the emerging brain circuits and cognitive functions to provide multi-dimensional training for school children.

Hence, cognitive enrichment based on age-specific developmental abilities and providing supplementary skill training to enhance individual cognitive skills can strengthen knowledge acquisition and organization. Potentially important factors for enriching the learning process are to provide a favorable environment that enhances experience-dependent plasticity, which includes positive classroom interactions, home environment, and socio-emotional relationships.

## Author Contributions

Both authors declare that the intellectual contribution and efforts were equally shared while conducting this review and they approve this for publication.

## Conflict of Interest

The authors declare that the research was conducted in the absence of any commercial or financial relationships that could be construed as a potential conflict of interest.

## Publisher’s Note

All claims expressed in this article are solely those of the authors and do not necessarily represent those of their affiliated organizations, or those of the publisher, the editors and the reviewers. Any product that may be evaluated in this article, or claim that may be made by its manufacturer, is not guaranteed or endorsed by the publisher.
